# Supplementary material for: A longitudinal study of the pulmonary mycobiome in subjects with and without chronic obstructive pulmonary disease
Source: PLoS One. 2022 May 12;17(5):e0267195. doi: 10.1371/journal.pone.0267195 (PMC9098062; doi:10.1371/journal.pone.0267195)
Supplement: S1 Fig — Most abundant fungal taxonomic assignments in (A) oral wash and (B) bronchoalveolar lavage. AB: Intercurrent antibiotic use, First: first bronchoscopy, Second: second bronchoscopy. Samples are sorted on time between bronchoscopies. Each column represents a sample, and columns from the first bronchoscopy and the second bronchoscopy correspond to each other. That means, a first bronchoscopy column above and the corresponding second bronchoscopy column below show samples from the same participant. Not all sequences could be assigned taxonomy at the genus level and are therefore displayed as o__Malasseziales, o__Tremellales, or p__Ascomycota. (PDF) [file pone.0267195.s001.pdf]

A longitudinal study of the pulmonary mycobiome in subjects with and without chronic obstructive pulmonary disease

Supporting Information, S1 Fig

Einar M. H. Martinsen[1*], Tomas M. L. Eagan[1,2], Harald G. Wiker HG[1,3], Elise O. Leiten[1], Gunnar R. Husebø[1,2], Kristel S. Knudsen[2], Solveig Tangedal[1,2], Walter Sanseverino[4], Andreu Paytuví-Gallart[4], and Rune Nielsen[1,2]

[1]Department of Clinical Science, University of Bergen, Bergen, Norway

[2]Department of Thoracic Medicine, Haukeland University Hospital, Bergen, Norway

[3]Department of Microbiology, Haukeland University Hospital, Bergen, Norway

[4]Sequentia Biotech SL, Barcelona, Spain

* Corresponding author

E-mail: einar.martinsen@uib.no

S1 Fig. Most abundant fungal taxonomic assignments in (A) oral wash and (B) bronchoalveolar lavage.

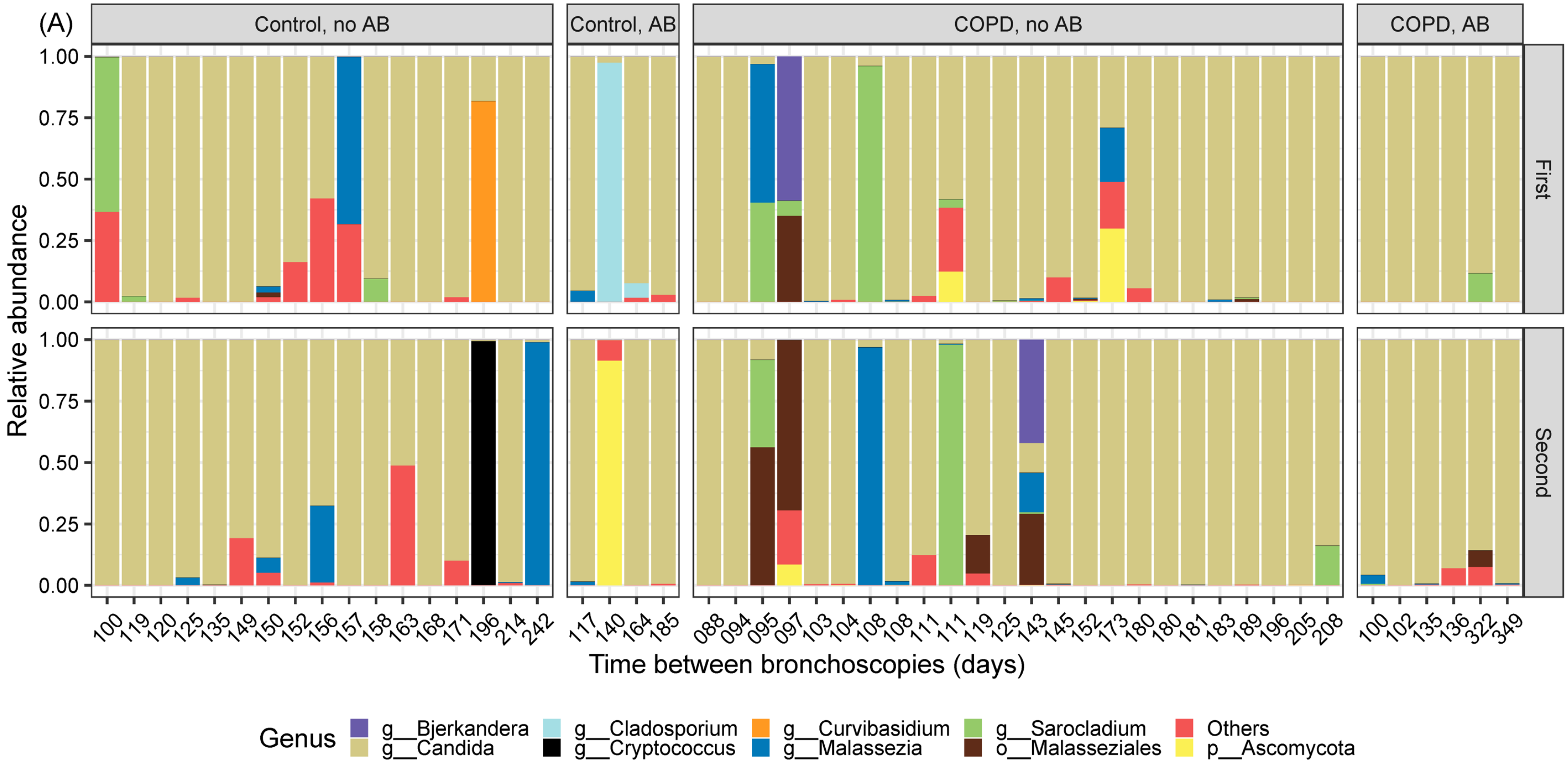

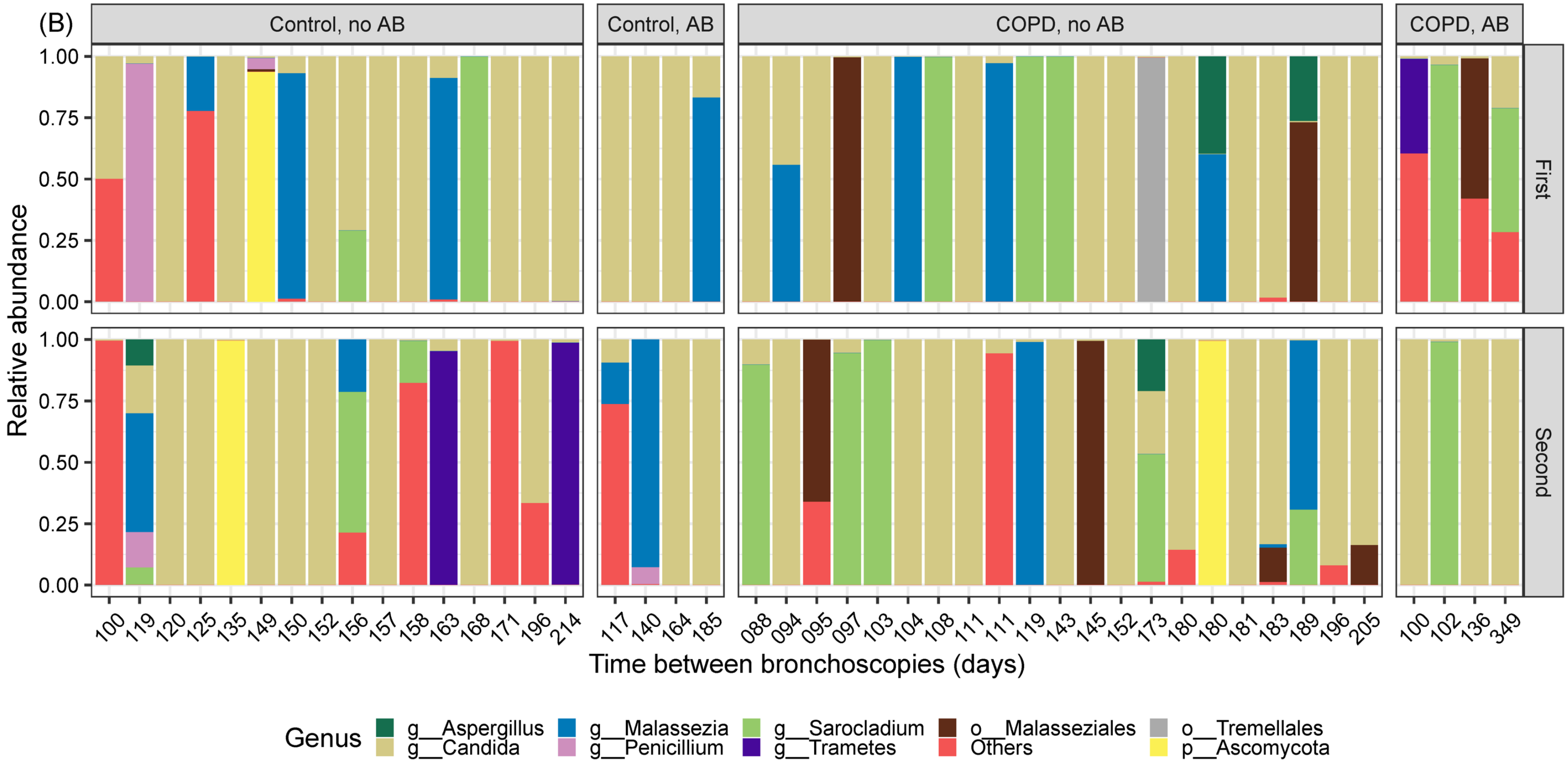

AB: Intercurrent antibiotic use, First: first bronchoscopy, Second: second bronchoscopy. Samples are sorted on time between bronchoscopies. Each column represents a sample, and columns from the first bronchoscopy and the second bronchoscopy corresponds to each other. That means, a first bronchoscopy column above and the corresponding second bronchoscopy column below show samples from the same participant. Not all sequences could be assigned taxonomy at the genus level and are therefore displayed as *o__Malasseziales*, *o__Tremellales*, or *p__Ascomycota*.
