# Supplementary material for: A longitudinal study of the pulmonary mycobiome in subjects with and without chronic obstructive pulmonary disease
Source: PLoS One. 2022 May 12;17(5):e0267195. doi: 10.1371/journal.pone.0267195 (PMC9098062; doi:10.1371/journal.pone.0267195)
Supplement: S2 Fig — Yue-Clayton plots of (A) oral wash and (B) bronchoalveolar lavage sample pairs from participants examined twice in the MicroCOPD study. YC: Yue-Clayton measure, First: first bronchoscopy, Second: Second bronchoscopy. A Yue-Clayton measure of 0 means identical sample pairs, while a Yue-Clayton measure of 1 means unidentical sample pairs. (PDF) [file pone.0267195.s002.pdf]

A longitudinal study of the pulmonary mycobiome in subjects with and without chronic obstructive pulmonary disease

Supporting Information, S2 Fig

Einar M. H. Martinsen[1*], Tomas M. L. Eagan[1,2], Harald G. Wiker HG[1,3], Elise O. Leiten[1], Gunnar R. Husebø[1,2], Kristel S. Knudsen[2], Solveig Tangedal[1,2], Walter Sanseverino[4], Andreu Paytuví-Gallart[4], and Rune Nielsen[1,2]

[1]Department of Clinical Science, University of Bergen, Bergen, Norway

[2]Department of Thoracic Medicine, Haukeland University Hospital, Bergen, Norway

[3]Department of Microbiology, Haukeland University Hospital, Bergen, Norway

[4]Sequentia Biotech SL, Barcelona, Spain

* Corresponding author

E-mail: einar.martinsen@uib.no

S2 Fig. Yue-Clayton plots of (A) oral wash and (B) bronchoalveolar lavage sample pairs from participants examined twice in the MicroCOPD study.

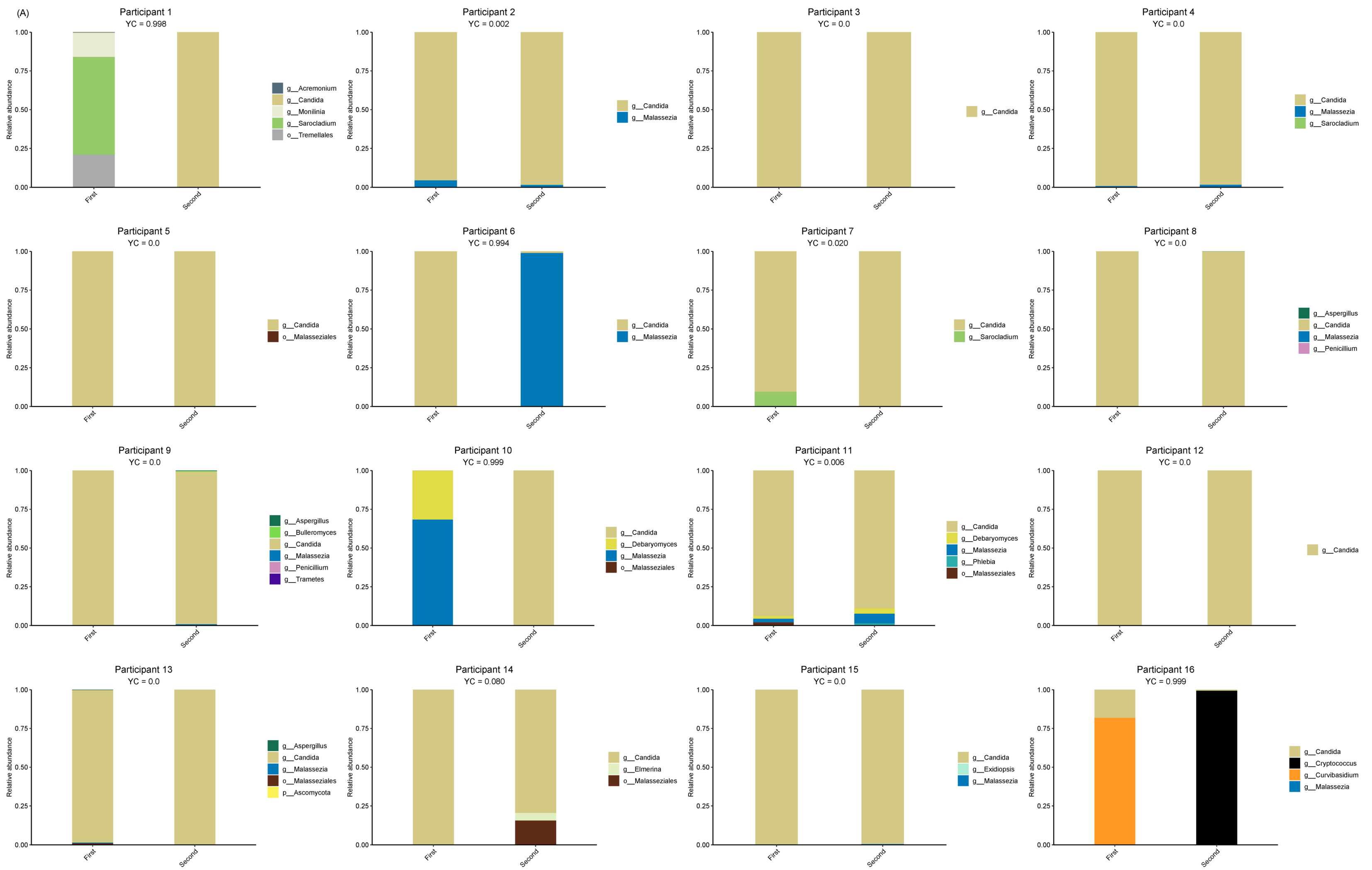

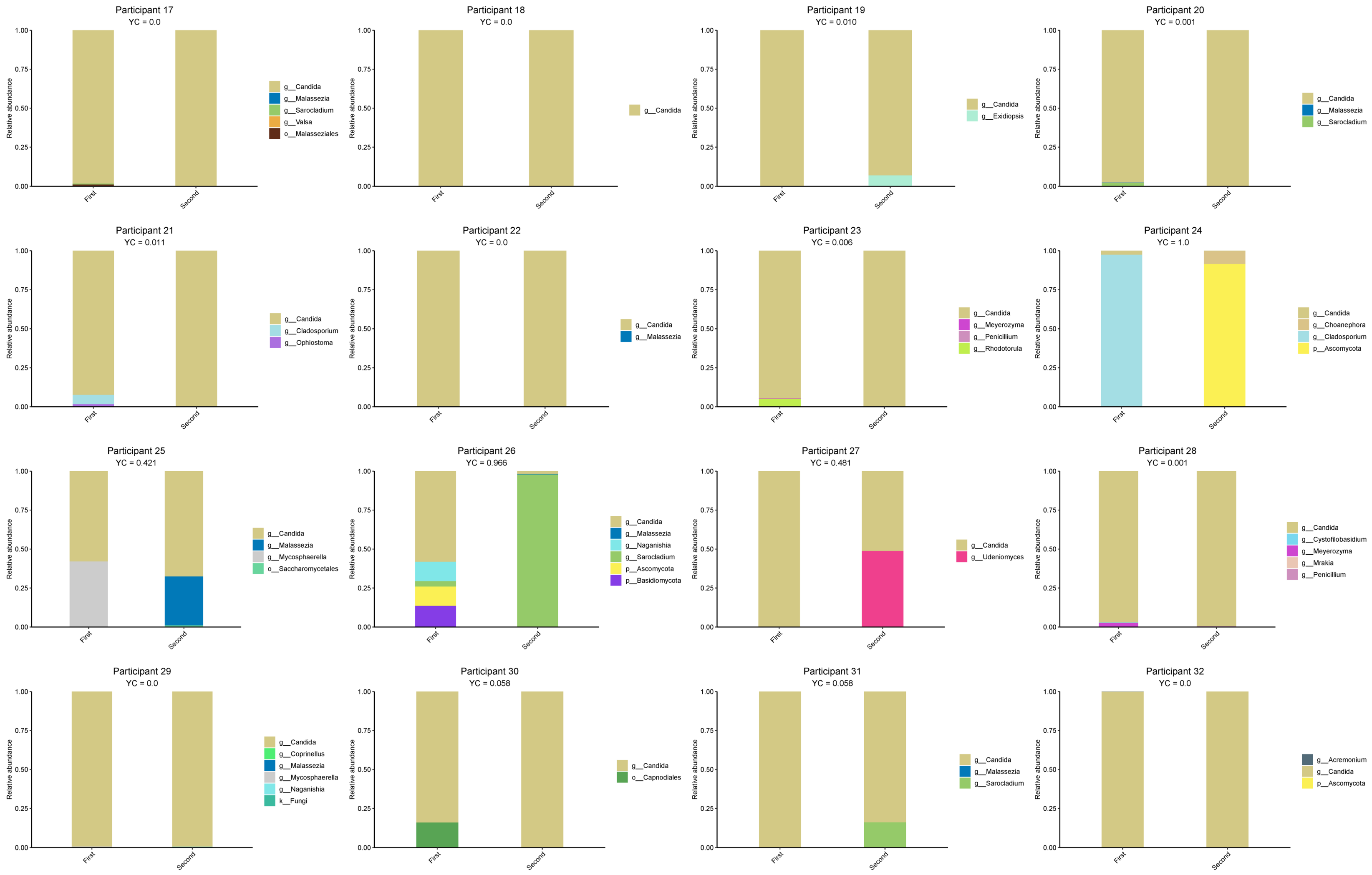
Participant 17
YC = 0.0
g__Candida
g__Malassezia
g__Sarocladium
g__Valsa
o__Malasseziales
Participant 18
YC = 0.0
g__Candida
Participant 19
YC = 0.010
g__Candida
g__Exidiopsis
Participant 20
YC = 0.001
g__Candida
g__Malassezia
g__Sarocladium
Participant 21
YC = 0.011
g__Candida
g__Cladosporium
g__Ophiostoma
Participant 22
YC = 0.0
g__Candida
g__Malassezia
Participant 23
YC = 0.006
g__Candida
g__Meyerozyma
g__Penicillium
g__Rhodotorula
Participant 24
YC = 1.0
g__Candida
g__Choanephora
g__Cladosporium
p__Ascomycota
Participant 25
YC = 0.421
g__Candida
g__Malassezia
g__Mycosphaerella
o__Saccharomycetales
Participant 26
YC = 0.966
g__Candida
g__Malassezia
g__Naganishia
g__Sarocladium
p__Ascomycota
p__Basidiomycota
Participant 27
YC = 0.481
g__Candida
g__Udeniomyces
Participant 28
YC = 0.001
g__Candida
g__Cystofilobasidium
g__Meyerozyma
g__Mrakia
g__Penicillium
Participant 29
YC = 0.0
g__Candida
g__Coprinellus
g__Malassezia
g__Mycosphaerella
g__Naganishia
k__Fungi
Participant 30
YC = 0.058
g__Candida
o__Capnodiales
Participant 31
YC = 0.058
g__Candida
g__Malassezia
g__Sarocladium
Participant 32
YC = 0.0
g__Acremonium
g__Candida
p__Ascomycota
Relative abundance
0.00
0.25
0.50
0.75
1.00
First
Second

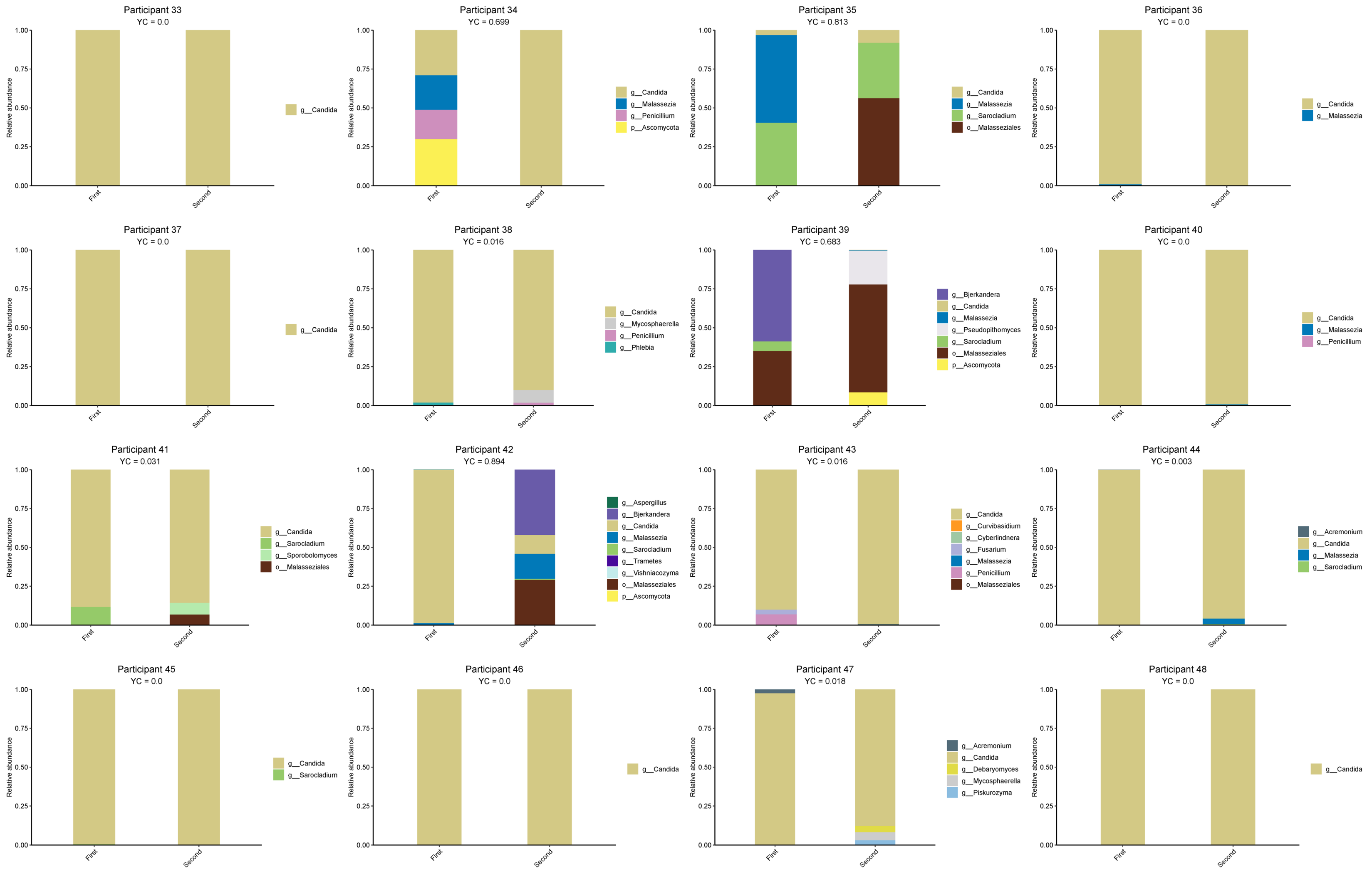

Participant 33
YC = 0.0
g__Candida
Participant 34
YC = 0.699
g__Candida
g__Malassezia
g__Penicillium
p__Ascomycota
Participant 35
YC = 0.813
g__Candida
g__Malassezia
g__Sarocladium
o__Malasseziales
Participant 36
YC = 0.0
g__Candida
g__Malassezia
Participant 37
YC = 0.0
g__Candida
Participant 38
YC = 0.016
g__Candida
g__Mycosphaerella
g__Penicillium
g__Phlebia
Participant 39
YC = 0.683
g__Bjerkandera
g__Candida
g__Malassezia
g__Pseudopithomyces
g__Sarocladium
o__Malasseziales
p__Ascomycota
Participant 40
YC = 0.0
g__Candida
g__Malassezia
g__Penicillium
Participant 41
YC = 0.031
g__Candida
g__Sarocladium
g__Sporobolomyces
o__Malasseziales
Participant 42
YC = 0.894
g__Aspergillus
g__Bjerkandera
g__Candida
g__Malassezia
g__Sarocladium
g__Trametes
g__Vishniacozyma
o__Malasseziales
p__Ascomycota
Participant 43
YC = 0.016
g__Candida
g__Curvibasidium
g__Cyberlindnera
g__Fusarium
g__Malassezia
g__Penicillium
o__Malasseziales
Participant 44
YC = 0.003
g__Acremonium
g__Candida
g__Malassezia
g__Sarocladium
Participant 45
YC = 0.0
g__Candida
g__Sarocladium
Participant 46
YC = 0.0
g__Candida
Participant 47
YC = 0.018
g__Acremonium
g__Candida
g__Debaryomyces
g__Mycosphaerella
g__Piskurozyma
Participant 48
YC = 0.0
g__Candida
Relative abundance
1.00
0.75
0.50
0.25
0.00
First
Second

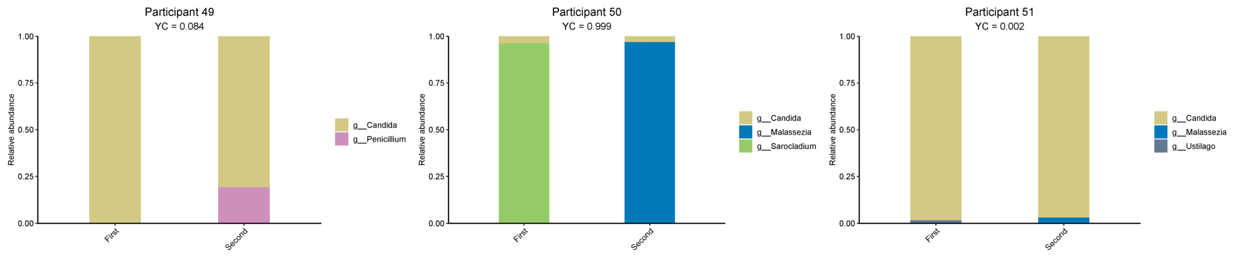
Participant 49
YC = 0.084
Relative abundance
1.00
0.75
0.50
0.25
0.00
First
Second
g__Candida
g__Penicillium
Participant 50
YC = 0.999
Relative abundance
1.00
0.75
0.50
0.25
0.00
First
Second
g__Candida
g__Malassezia
g__Sarocladium
Participant 51
YC = 0.002
Relative abundance
1.00
0.75
0.50
0.25
0.00
First
Second
g__Candida
g__Malassezia
g__Ustilago

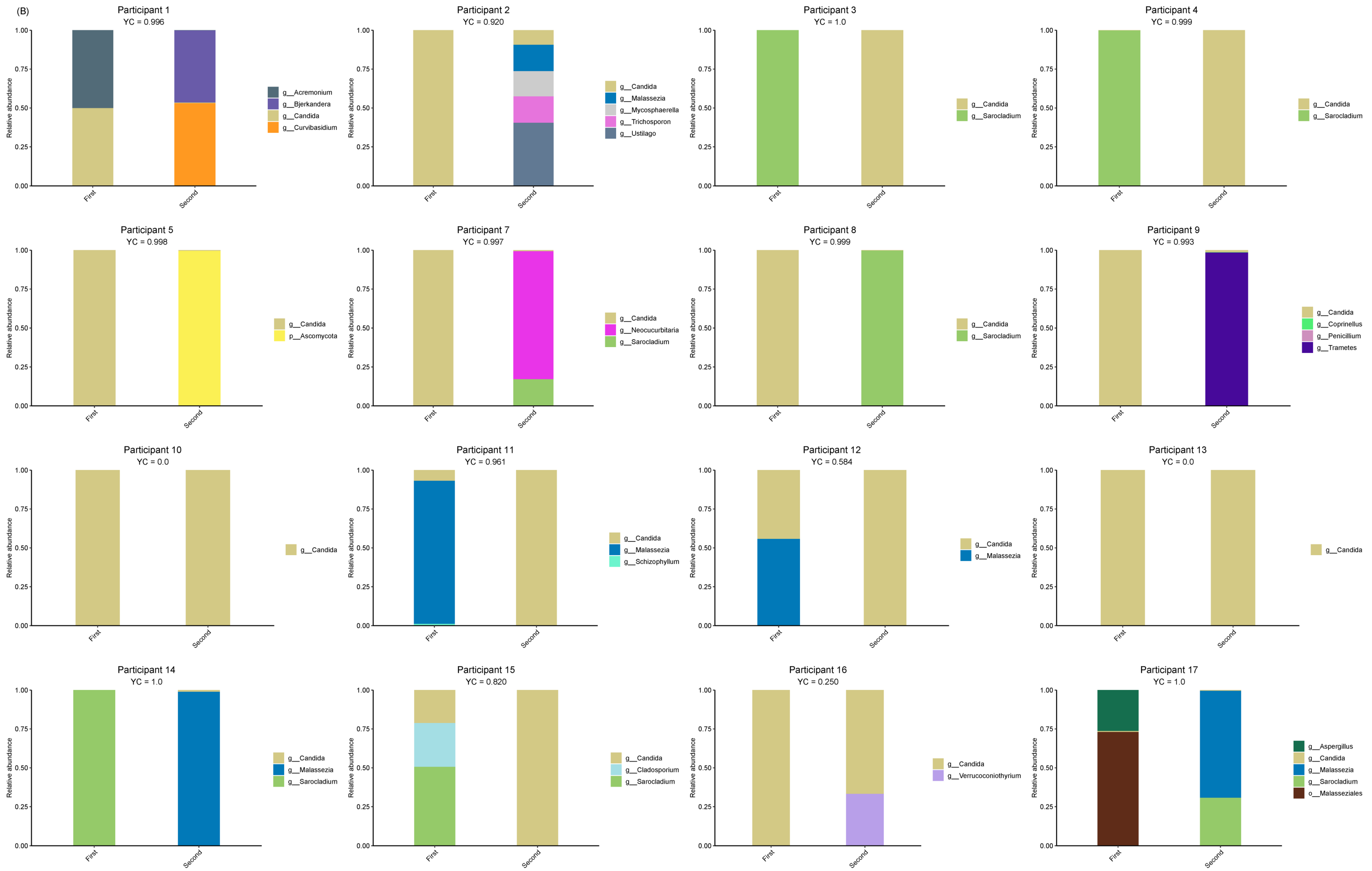

(B)
Participant 1
YC = 0.996
g__Acremonium
g__Bjerkandera
g__Candida
g__Curvibasidium
Participant 2
YC = 0.920
g__Candida
g__Malassezia
g__Mycosphaerella
g__Trichosporon
g__Ustilago
Participant 3
YC = 1.0
g__Candida
g__Sarocladium
Participant 4
YC = 0.999
g__Candida
g__Sarocladium
Participant 5
YC = 0.998
g__Candida
p__Ascomycota
Participant 7
YC = 0.997
g__Candida
g__Neocucurbitaria
g__Sarocladium
Participant 8
YC = 0.999
g__Candida
g__Sarocladium
Participant 9
YC = 0.993
g__Candida
g__Coprinellus
g__Penicillium
g__Trametes
Participant 10
YC = 0.0
g__Candida
Participant 11
YC = 0.961
g__Candida
g__Malassezia
g__Schizophyllum
Participant 12
YC = 0.584
g__Candida
g__Malassezia
Participant 13
YC = 0.0
g__Candida
Participant 14
YC = 1.0
g__Candida
g__Malassezia
g__Sarocladium
Participant 15
YC = 0.820
g__Candida
g__Cladosporium
g__Sarocladium
Participant 16
YC = 0.250
g__Candida
g__Verrucoconiothyrium
Participant 17
YC = 1.0
g__Aspergillus
g__Candida
g__Malassezia
g__Sarocladium
o__Malasseziales
Relative abundance
0.00
0.25
0.50
0.75
1.00
First
Second

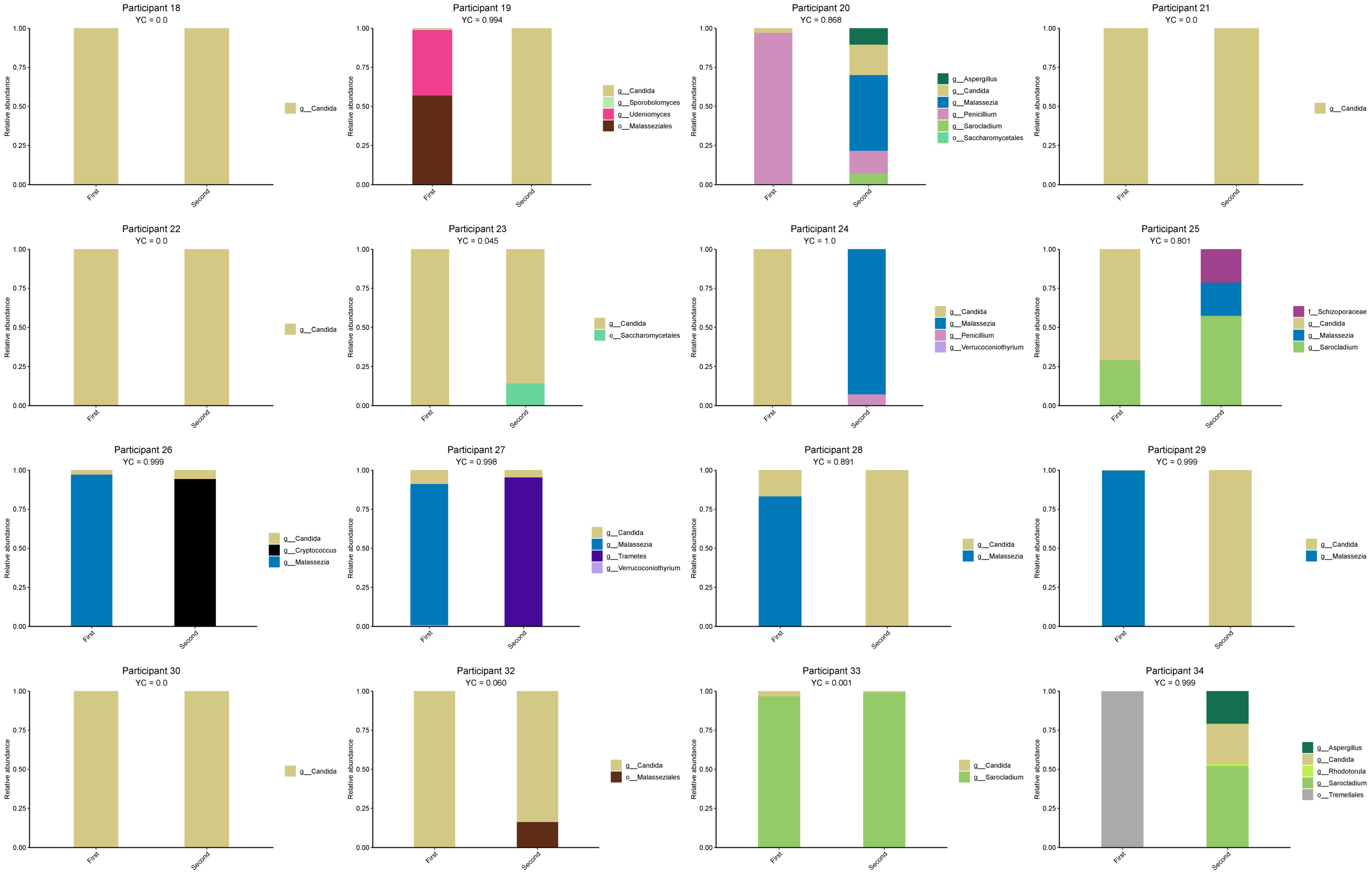
Participant 18
YC = 0.0
g__Candida
Participant 19
YC = 0.994
g__Candida
g__Sporobolomyces
g__Udeniomyces
o__Malasseziales
Participant 20
YC = 0.868
g__Aspergillus
g__Candida
g__Malassezia
g__Penicillium
g__Sarocladium
o__Saccharomycetales
Participant 21
YC = 0.0
g__Candida
Participant 22
YC = 0.0
g__Candida
Participant 23
YC = 0.045
g__Candida
o__Saccharomycetales
Participant 24
YC = 1.0
g__Candida
g__Malassezia
g__Penicillium
g__Verrucoconiothyrium
Participant 25
YC = 0.801
f__Schizoporaceae
g__Candida
g__Malassezia
g__Sarocladium
Participant 26
YC = 0.999
g__Candida
g__Cryptococcus
g__Malassezia
Participant 27
YC = 0.998
g__Candida
g__Malassezia
g__Trametes
g__Verrucoconiothyrium
Participant 28
YC = 0.891
g__Candida
g__Malassezia
Participant 29
YC = 0.999
g__Candida
g__Malassezia
Participant 30
YC = 0.0
g__Candida
Participant 32
YC = 0.060
g__Candida
o__Malasseziales
Participant 33
YC = 0.001
g__Candida
g__Sarocladium
Participant 34
YC = 0.999
g__Aspergillus
g__Candida
g__Rhodotorula
g__Sarocladium
o__Tremellales
Relative abundance
First
Second

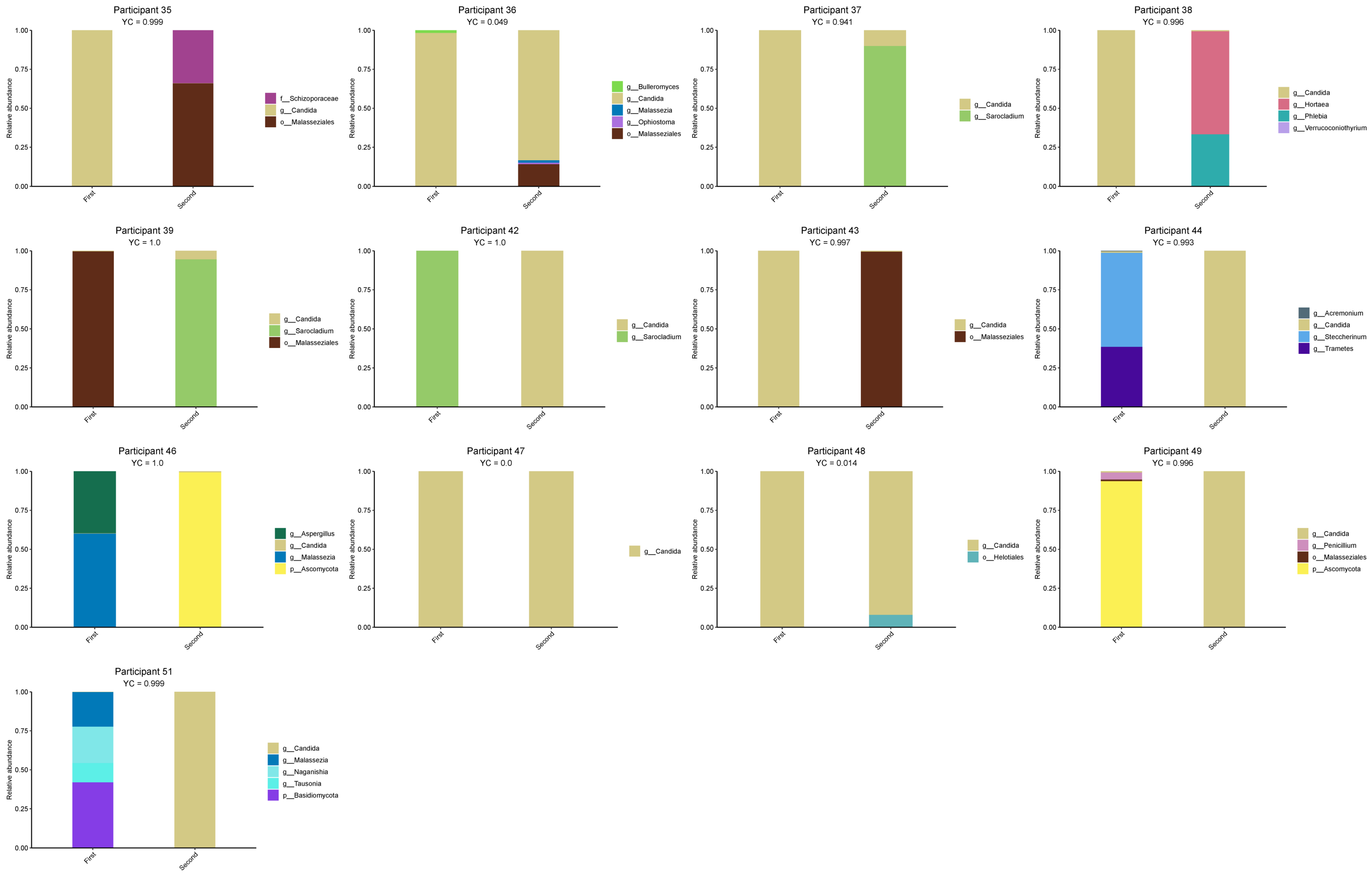

YC: Yue-Clayton measure, First: first bronchoscopy, Second: Second bronchoscopy. A Yue-Clayton measure of 0 means identical sample pairs, while a Yue-Clayton measure of 1 means unidentical sample pairs.
