# Supplementary material for: A longitudinal study of the pulmonary mycobiome in subjects with and without chronic obstructive pulmonary disease
Source: PLoS One. 2022 May 12;17(5):e0267195. doi: 10.1371/journal.pone.0267195 (PMC9098062; doi:10.1371/journal.pone.0267195)
Supplement: S3 Fig — Principal coordinates analysis plot using (A) Bray-Curtis and (B) Jaccard coloured by bronchoscopy number and sample type. First BAL: bronchoalveolar lavage samples from first bronchoscopy, First OW: oral wash samples from first bronchoscopy, Second BAL: bronchoalveolar lavage samples from second bronchoscopy, Second OW: oral wash samples from second bronchoscopy. (PDF) [file pone.0267195.s003.pdf]

A longitudinal study of the pulmonary mycobiome in subjects with and without chronic obstructive pulmonary disease

Supporting Information, S3 Fig

Einar M. H. Martinsen[1*], Tomas M. L. Eagan[1,2], Harald G. Wiker HG[1,3], Elise O. Leiten[1], Gunnar R. Husebø[1,2], Kristel S. Knudsen[2], Solveig Tangedal[1,2], Walter Sanseverino[4], Andreu Paytuví-Gallart[4], and Rune Nielsen[1,2]

[1]Department of Clinical Science, University of Bergen, Bergen, Norway

[2]Department of Thoracic Medicine, Haukeland University Hospital, Bergen, Norway

[3]Department of Microbiology, Haukeland University Hospital, Bergen, Norway

[4]Sequentia Biotech SL, Barcelona, Spain

* Corresponding author

E-mail: einar.martinsen@uib.no

S3 Fig. Principal coordinates analysis plot using (A) Bray-Curtis and (B) Jaccard coloured by bronchoscopy number and sample type.

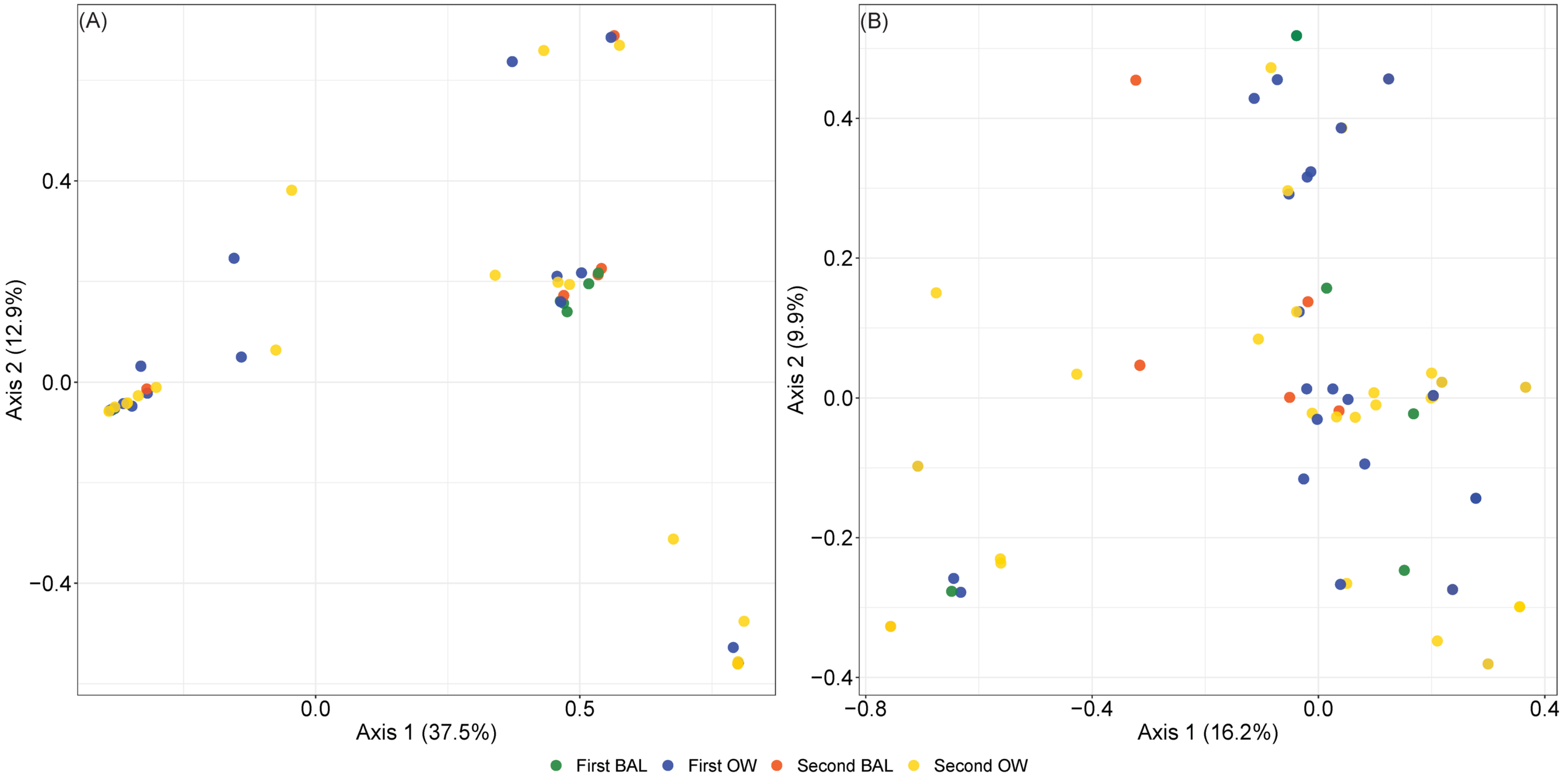

First BAL: bronchoalveolar lavage samples from first bronchoscopy, First OW: oral wash samples from first bronchoscopy, Second BAL: bronchoalveolar lavage samples from second bronchoscopy, Second OW: oral wash samples from second bronchoscopy.
