# Supplementary material for: A longitudinal study of the pulmonary mycobiome in subjects with and without chronic obstructive pulmonary disease
Source: PLoS One. 2022 May 12;17(5):e0267195. doi: 10.1371/journal.pone.0267195 (PMC9098062; doi:10.1371/journal.pone.0267195)
Supplement: S7 Fig — Principal coordinates analysis plot of BAL samples using (A and C) Bray-Curtis and (B and D) Jaccard coloured by bronchoscopy number and intercurrent antibiotic use with and without lines. First: first bronchoscopy, Second: Second bronchoscopy. A line was drawn between samples from the same participant. Red lines refer to participants receiving intercurrent antibiotics, while green lines refer to participants not receiving intercurrent antibiotics. (PDF) [file pone.0267195.s007.pdf]

A longitudinal study of the pulmonary mycobiome in subjects with and without chronic obstructive pulmonary disease

Supporting Information, S7 Fig

Einar M. H. Martinsen[1*], Tomas M. L. Eagan[1,2], Harald G. Wiker HG[1,3], Elise O. Leiten[1], Gunnar R. Husebø[1,2], Kristel S. Knudsen[2], Solveig Tangedal[1,2], Walter Sanseverino[4], Andreu Paytuví-Gallart[4], and Rune Nielsen[1,2]

[1]Department of Clinical Science, University of Bergen, Bergen, Norway

[2]Department of Thoracic Medicine, Haukeland University Hospital, Bergen, Norway

[3]Department of Microbiology, Haukeland University Hospital, Bergen, Norway

[4]Sequentia Biotech SL, Barcelona, Spain

* Corresponding author

E-mail: einar.martinsen@uib.no

S7 Fig. Principal coordinates analysis plot of BAL samples using (A and C) Bray-Curtis and (B and D) Jaccard coloured by bronchoscopy number and intercurrent antibiotic use with and without lines.

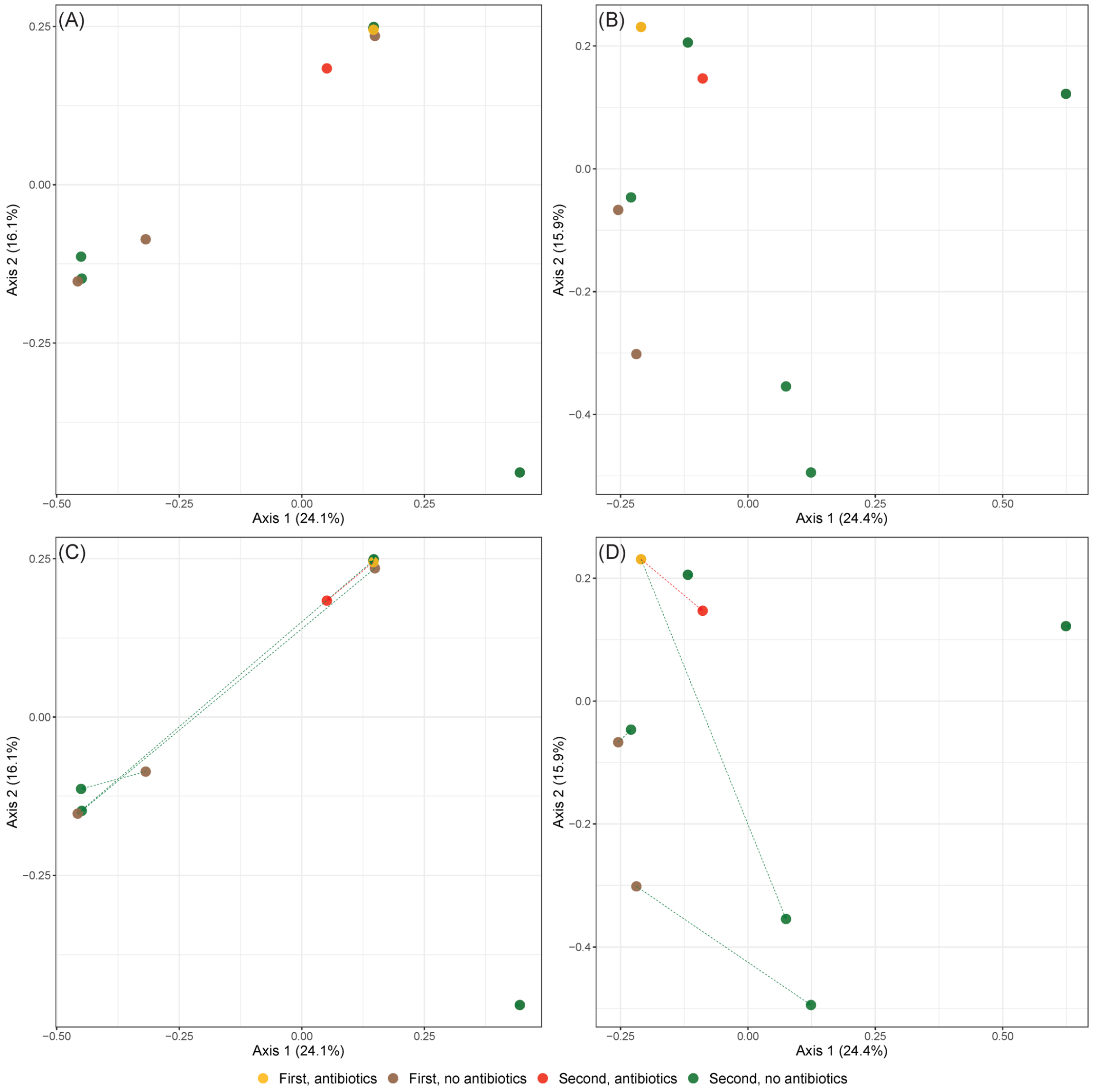

First: first bronchoscopy, Second: Second bronchoscopy. A line was drawn between samples from the same participant. Red lines refer to participants receiving intercurrent antibiotics, while green lines refer to participants not receiving intercurrent antibiotics.
