# Supplementary material for: A longitudinal study of the pulmonary mycobiome in subjects with and without chronic obstructive pulmonary disease
Source: PLoS One. 2022 May 12;17(5):e0267195. doi: 10.1371/journal.pone.0267195 (PMC9098062; doi:10.1371/journal.pone.0267195)
Supplement: Attachment — Submitted filename: response_to_reviewers_20Nov2021.docx [file pone.0267195.s009.docx]

Haugesund 20^th^ of November 2021

Concerning manuscript PONE-D-21-19426, *A longitudinal study of the pulmonary mycobiome in subjects with and without chronic obstructive pulmonary disease*.

**Dear Academic Editor Christine M. Freeman**

Thank you for the valuable comments to our paper, and for giving us the possibility to submit a revised version. The original comments of the reviewers and our responses follow. We have added numbering to the comments (C) and our answers (A) to enhance reading and further correspondence.

Editor’s comments:

C1	Thank you for submitting your manuscript to PLOS ONE. After careful consideration, we feel that it has merit but does not fully meet PLOS ONE’s publication criteria as it currently stands. Therefore, we invite you to submit a revised version of the manuscript that addresses the points raised during the review process.

A1	We hope that we have been able to address all comments from the reviewers, and that these comments have led to improvement of the manuscript.

C2	Please ensure that your manuscript meets PLOS ONE's style requirements, including those for file naming. The PLOS ONE style templates can be found at

https://journals.plos.org/plosone/s/file?id=wjVg/PLOSOne_formatting_sample_main_body.pdf and https://journals.plos.org/plosone/s/file?id=ba62/PLOSOne_formatting_sample_title_aauthor_affiliations.pdf.

A2	We have reviewed our submitted paper and believe our formatting is in line with the journal requirements.

C3	Please provide additional details regarding participant consent. In the ethics statement in the Methods and online submission information, please ensure that you have specified whether consent was informed."

A3	We have now elaborated on the ethics statement (line 90 in revised manuscript) and have specifically included that consent was informed.

C4	Thank you for stating the following in the Competing Interests/Financial Disclosure* (delete as necessary) section:

“Einar M. H. Martinsen, Elise O. Leiten, Gunnar Husebø, and Solveig Tangedal declare no conflict of interest. Walter Sanseverino and Andreu Paytuví-Gallart are employed at Sequentia Biotech SL. Tomas M. L. Eagan reports lecture fees from Boehringer and AstraZeneca, and grants from GlaxoSmithKline outside the submitted work. Harald G. Wiker reports being head of the educational programme for medicine at the University of Bergen. Kristel S. Knudsen reports lecture fees from Boehringer Ingelheim and Roche. Rune Nielsen reports grants from Boehringer Ingelheim, GlaxoSmithKline, AstraZeneca, and the Timber Merchant Delphins Endowment, in addition to lecture fees from GlaxoSmithKline and AstraZeneca. Rune Nielsen also reports being unpaid member of The Norwegian Respiratory Society, unpaid GOLD national delegate, unpaid ERS national delegate, and paid member of the Reference group for new Norwegian guidelines for COPD (The Norwegian Directorate of Health). This does not alter our adherence to PLOS ONE policies on sharing data and materials.”

We note that one or more of the authors are employed by a commercial company: GlaxoSmithKline,Boehringer and AstraZeneca,

Please provide an amended Funding Statement declaring this commercial affiliation, as well as a statement regarding the Role of Funders in your study. If the funding organization did not play a role in the study design, data collection and analysis, decision to publish, or preparation of the manuscript and only provided financial support in the form of authors' salaries and/or research materials, please review your statements relating to the author contributions, and ensure you have specifically and accurately indicated the role(s) that these authors had in your study. You can update author roles in the Author Contributions section of the online submission form.

A4	The specific authors were not employed by the companies but received grants and lecture fees. Their roles are stated in the Author contribution section. I could not find any possibility to change the Author contribution section of the online submission form. This information is therefore included in the revised manuscript.

C5	Please also include the following statement within your amended Funding Statement.

“The funder provided support in the form of salaries for authors [insert relevant initials], but did not have any additional role in the study design, data collection and analysis, decision to publish, or preparation of the manuscript. The specific roles of these authors are articulated in the ‘author contributions’ section.”

If your commercial affiliation did play a role in your study, please state and explain this role within your updated Funding Statement.

A5	We believe the requested information was already included in the Funding statement. Only Sequentia Biotech provided support in the form of salaries, and this is stated.

C6	Please also provide an updated Competing Interests Statement declaring this commercial affiliation along with any other relevant declarations relating to employment, consultancy, patents, products in development, or marketed products, etc.

Within your Competing Interests Statement, please confirm that this commercial affiliation does not alter your adherence to all PLOS ONE policies on sharing data and materials by including the following statement: "This does not alter our adherence to PLOS ONE policies on sharing data and materials.” (as detailed online in our guide for authors http://journals.plos.org/plosone/s/competing-interests) . If this adherence statement is not accurate and there are restrictions on sharing of data and/or materials, please state these. Please note that we cannot proceed with consideration of your article until this information has been declared.

Please include both an updated Funding Statement and Competing Interests Statement in your cover letter. We will change the online submission form on your behalf.

A6	The requested information was already included in the Competing interests statement. We believe it is correct as is, and we have not done any further amendments.

C7	We noted in your submission details that a portion of your manuscript may have been presented or published elsewhere. Please clarify whether this [conference proceeding or publication] was peer-reviewed and formally published. If this work was previously peer-reviewed and published, in the cover letter please provide the reason that this work does not constitute dual publication and should be included in the current manuscript.

A7	The draft has been included in the corresponding author’s thesis, which was submitted for evaluation at the University of Bergen 17th of March 2021 and approved by a committee consisting of two external members and one internal member. The thesis is published under open access here: <https://bora.uib.no/bora-xmlui/handle/11250/2760320>.

The current manuscript was included in the thesis submitted for evaluation and discussed in the published thesis, but the actual manuscript was omitted from the published thesis.

C8	We note that you are reporting an analysis of a microarray, next-generation sequencing, or deep sequencing data set. PLOS requires that authors comply with field-specific standards for preparation, recording, and deposition of data in repositories appropriate to their field. Please upload these data to a stable, public repository (such as ArrayExpress, Gene Expression Omnibus (GEO), DNA Data Bank of Japan (DDBJ), NCBI GenBank, NCBI Sequence Read Archive, or EMBL Nucleotide Sequence Database (ENA)). In your revised cover letter, please provide the relevant accession numbers that may be used to access these data. For a full list of recommended repositories, see http://journals.plos.org/plosone/s/data-availability#loc-omics or http://journals.plos.org/plosone/s/data-availability#loc-sequencing.

A8	The sequencing data are deposited in the DRYAD repository with accession link: https://datadryad.org/stash/share/GyTywoV1CQcB1aZsToAeTtDjtaiW_

sz0dSHSSORMEQ

If the paper is accepted, the data will be freely accessible with the following link: https://doi.org/10.5061/dryad.kd51c5b41

C9	Please review your reference list to ensure that it is complete and correct. If you have cited papers that have been retracted, please include the rationale for doing so in the manuscript text, or remove these references and replace them with relevant current references. Any changes to the reference list should be mentioned in the rebuttal letter that accompanies your revised manuscript. If you need to cite a retracted article, indicate the article’s retracted status in the References list and also include a citation and full reference for the retraction notice.

A9	We have reviewed the reference list during the revision process. We identified no paper that was retracted.

**Reviewer 1**

C10	This is a very well written manuscript that reports valuable information relative to the temporal stability of the human lung mycobiome.

I have only a few suggestions for improvement:

Lines 213 and 495. Change “corresponds” to “correspond”

A10	Thank you for making us aware of this misspelling. We have changed as suggested in the revised manuscripts (line 216 and 509 in revised manuscript).

C11	Legends for figures 3 and 4A. I suggest adding to the legend information regarding the significance of the statistical values that are shown on the brackets on the figures.

A11	We have now added additional information of the chosen significance level.

C12	I suggest an expanded explanation of intercurrent antibiotic use in Materials and Methods. This section currently states that recent antibiotic use was a reason for postponing participation in the study. But the fact that some participants then had antibiotic treatment becomes a variable in the study with no real discussion of the antibiotics involved or the timeline of their use.

A12	We appreciate the suggestion and have added more information on intercurrent antibiotic use in the Materials and methods section (line 86 and 95-98 in revised manuscript).

C13	Line 318. Change “was” to “were”.

A13	Thank you for making us aware of this misspelling. We have changed as suggested in the revised manuscript.

C14	Line 320. Perhaps change “paper” to “study”

A14	We believe it is more correct to use “paper” in this case as we have published several findings from the study while the observation mentioned in this sentence is specific for this paper. However, we added “fungal” in front of alpha diversity for clarity (line 327 in revised manuscript).

C15	Line 321. Perhaps change “but were” to “but it was”

A15	We agree. Changed as suggested in the revised manuscript.

C16	There is a recent paper by Rubio-Portillo et al. that compares the lung mycobiome to the fungal community found in the home environment. The authors might want to see if this study is relevant to the work presented in the current manuscript. [Rubio-Portillo et al. Microorganisms 8, 1717 (2020)].

A16	We would like to thank the reviewer for the suggested revisions. The suggested paper examines an interesting association and add important data on the research of the lower respiratory tract mycobiome. We believe, however, that the study’s relevance is not directly comparable to our paper. There are several studies investigating the lower respiratory mycobiome, but few looking at the mycobiomes stability which is the aim in the current paper. We have chosen not to include the suggested paper as our paper specifically examine mycobiome stability but are grateful for an interesting read!

**Reviewer 2**

C17	The goal of this study is to analyze the stability of the mycobiome in BAL and oral wash over time in both healthy participants and participants without COPD. Repeat bronchoscopies were performed between 3 and 12 months. Mycobiome data from the oral wash and broncholaveolar lavage have been published, but this study is focusing on the stability of the mycobiome and therefore has a different scientific question and purpose.

The statistical analysis is appropriate and the data support the conclusions. The manuscript is clearly written.

Based on the title and the abstract which states that "no apparent effect was seen on taxonomy from intercurrent antibiotic use or participant category", I expected to see analyses pertaining to the COPD status of the participant. I see that Figure 2 is separated into participant category, but where are the statistics that support the finding that there are no differences. I did not see this in the results.

A17	We agree that information in the abstract could be misleading. There are no analyses on the differences in the healthy lung mycobiome and the COPD mycobiome in the current paper as this was the focus of our previous mycobiome paper [1]. It is possible to visually examine differences by time in the different participant groups’ mycobiomes by looking at Fig 2, but the reviewer is right that no statistics were performed to support this. We have specified by adding “visually” in front of “seen” in our statements (in abstract and line 234 and 313 in revised manuscript).

The title refers to the inclusion and examination of longitudinal data from both subjects with and without COPD, not the comparison between these groups. Thus, we believe the title is descriptive.

C18	Page 5, Line 116 states that "the second portion of the BAL was utilised for mycobiome analysis". Is there any reason to think that this could impact the results? Would the signal from the first portion have been more robust?

A18	We appreciate input on this issue. As stated, only the second fraction of the BAL was used in the mycobiome analyses in the MicroCOPD study. Another paper by our research group has shown high similarity between the first and the second fraction of BAL, suggesting little impact from choice of fraction [2]. Furthermore, at least in theory, use of a second fraction could be a method to reduce impact from potential bronchoscopic carryover [3]. We have added some information on this in the discussion part of the manuscript (line 369-371 in revised manuscript).

C19	Table 1. Please include statistics for all demographics. This information is useful in evaluating the data.

A19	We agree, and have included this in the revised manuscript.

C20	Page 15, line 324. This information should be included in the results section prior to Figure 3. Please explain why there are almost 50 samples in Figure 2 and then only a handful of samples in Figure 3. All figure legends should include the number of participants or samples.

A20	Abundance data in a mycobiome study is not directly comparable. Each sample has its own sequencing depth or sum of abundances. Several techniques have been proposed to deal with this compositionality, including rarefaction (subsampling without replacement to a given sampling depth). We described the rarefaction process in the Materials and methods section (line 161 and 162 in revised manuscript). In short, the rarefaction subsamples each sample to a given rarefaction or sampling/sequencing depth without replacement. Samples with a sequence count below this value will be discarded. The sampling depth was set as high as possible while excluding a minimum of samples. Still, rarefaction omitted most BAL samples in the longitudinal paper (paper IV), leaving only 12 left for analyses and decreasing power in the diversity analyses including BAL samples. We admit that rarefaction normalise data at the expense of power loss. However, we used *QIIME 2* [4], one of the main pipelines used to perform microbiome analyses, and their diversity metric plugin or command has an included rarefaction step.

We agree that this information is better suited for the Results section and it has been moved in the revised manuscript (to line 249-250 in revised manuscript). We also reformulated the Discussion section to clarify on the issue (line 331-333 in revised manuscript).

Figure legends for Fig 2 and Fig 3 are updated with information on sample sizes. The information was included in the plot for Fig 4 and no changes were done to the figure legend.

C20	Did you consider evaluating inhaled corticosteroid usage or current vs former smoking status to see whether these variables had any influence on the mycobiome stability?

A20	This is an interesting suggestion and also something we considered. However, due to the low number of samples such sub-analyses seemed unfit, for instance due to the already mentioned rarefaction process. Furthermore, we have previously shown that the pulmonary mycobiomes do not seem to be influenced by smoking habits or use of inhaled steroids [1]. We included intercurrent antibiotics due to a potential influence on the stability through possible interactions with the bacterial microbiome. Further studies with a larger sample size are definitely warranted to examine effects on the mycobiome stability from both smoking and use of inhaled steroids.

C21	Page 12, line 258 -259: Please indicated that you are discussing S6B.

A21	Thanks for suggestion. Clarified in revised manuscript.

We hope that you find the revision satisfactory, but are of course happy to provide a second revision if necessary. All changes are marked with “track changes” function in the revised manuscript.

We thank the reviewers and the editor for helping improve our paper. Please do not hesitate to contact the corresponding author if unresolved issues remain.

On behalf of the writing group:

Einar Marius Hjellestad Martinsen

[einarmarius@haugnett.no](mailto:einar.martinsen@uib.no)

**References in response to reviewers**

1.	Martinsen EMH, Eagan TML, Leiten EO, Haaland I, Husebø GR, Knudsen KS, et al. The pulmonary mycobiome-A study of subjects with and without chronic obstructive pulmonary disease. PLoS One. 2021;16(4):e0248967.

2.	Grønseth R, Drengenes C, Wiker HG, Tangedal S, Xue Y, Husebø GR, et al. Protected sampling is preferable in bronchoscopic studies of the airway microbiome. ERJ Open Res. 2017;3(3).

3.	Charlson ES, Bittinger K, Haas AR, Fitzgerald AS, Frank I, Yadav A, et al.

Topographical continuity of bacterial populations in the healthy human respiratory tract.

Am J Respir Crit Care Med. 2011;184(8):957-63.

4.	Bolyen E, Rideout JR, Dillon MR, Bokulich NA, Abnet CC, Al-Ghalith GA, et al.

Reproducible, interactive, scalable and extensible microbiome data science using QIIME

2. Nat Biotechnol. 2019;37(8):852-7.
